# Pelvic mass in von Recklinghausen's neurofibromatosis: diagnostic issues: a case report

**DOI:** 10.1186/1757-1626-2-191

**Published:** 2009-11-12

**Authors:** Nicolas Kluger, Hélène Perrochia, Bernard Guillot

**Affiliations:** 1 Université Montpellier I, Service de Dermatologie, Hôpital Saint-Eloi, CHU de Montpellier, 80, avenue Augustin Fliche, FR-34295 Montpellier cedex 5, France; 2 Université Montpellier I, Service d'Anatomie et Cytologie Pathologiques, Hôpital Lapeyronie, CHU de Montpellier, France

## Abstract

**Background:**

Leiomyomas are benign smooth muscle cell tumours. They are the most common uterine neoplasms, although they may also occur elsewhere, such as in the gastrointestinal and urinary tracts. Leiomyomas are uncommon in von Recklinghausen's neurofibromatosis (NF1). However, the literature suggests that the association of NF1 and leiomyomas or leiomyosarcoma is not entirely coincidental.

**Case Report:**

We report here the unusual case of a 47-year-old woman with NF1 who presented menorrhagias and a hard, tender pelvic mass composed of uterine leiomyomas. Positron emission tomography/computed tomography disclosed an area of increased 18-fluorodeoxyglucose (FDG) uptake in the upper right part of the mass that raised the suspicion of malignancy. Magnetic resonance imaging revealed numerous intramural leiomyomas ranging from 1 to 6 cm, including a 6-cm submucosal leiomyoma that had abundant cellularity, matching FDG uptake. Abdominal hysterectomy was performed and microscopic examination confirmed the diagnosis of multiple benign smooth muscle tumours (uterine leiomyomatosis).

**Conclusion:**

Our case illustrates several diagnostic issues that arose while exploring this pelvic mass. Moreover, the association of uterine leiomyoma and NF1 may not be fortuitous.

## Introduction

Leiomyomas are benign smooth muscle cell tumours. They are the most common gynaecologic and uterine neoplasms, although they may also occur in other organs [1]. These tumours are an uncommon finding in von Recklinghausen's neurofibromatosis (NF1). The literature suggests, however, that the association of NF1 and leiomyomas or leiomyosarcoma may not be merely coincidental, as documented by various case reports [2,3]. In the context of NF1, leiomyomas mostly affect the gastrointestinal tract with multiple lesions, showing predominance in the proximal small bowel. We report here a patient with NF1 who presented with an abdominal mass that was found to be uterine leiomyomatosis. We particularly emphasise several diagnostic issues that arose while exploring this patient and discuss the potential association of the two conditions.

## Case Presentation

A 47-year-old woman was followed for sporadic NF1 diagnosed by the association of numerous café-au-lait spots, cutaneous neurofibromas (NFBs) of the limbs and trunk, nodular NFBs of the scalp, and a plexiform NFB of the ankle. Her medical history was notable for a thoracic paraspinal NFB extending into the neural foramina of T12-L1, which had been resected neurosurgically in 2005, and numerous cutaneous NFBs treated by CO2 laser sessions on a regular basis. During follow-up, a 10-cm, hard, tender pelvic mass was discovered that had been absent at the prior examination 12 months earlier. She acknowledged having experienced menorrhagias and pelvic pain and pressure for several weeks. Laboratory testing revealed decreased ferritin (3 ng/ml, N > 11) without anaemia and a mild inflammatory syndrome with a slightly elevated erythrocyte sedimentation rate at 30 mm, elevated fibrinogen of 5.5 mg/l (N < 4 mg/l), and C-reactive protein within the normal range. Transabdominal ultrasound imaging displayed numerous leiomyomas with a normal endometrium. Positron emission tomography/computed tomography (PET/CT) disclosed an area of increased 18-fluorodeoxyglucose (FDG) uptake in the upper right part of the pelvic mass. Magnetic resonance imaging (MRI) was then performed to evaluate the diagnosis of uterine leiomyomatosis, to map the lesions, and to rule out the diagnosis of NFB or malignant lesion-like sarcoma. MRI confirmed numerous intramural leiomyomas ranging in size from 1 to 6 cm. A 6-cm submucosal leiomyoma displayed abundant cellularity, matching the FDG uptake on PET/CT. The endometrium and ovaries were normal. The patient subsequently underwent abdominal hysterectomy without salpingo-oophorectomy to rule out a malignant lesion. The specimen consisted of a uterus with attached cervix. The uterus measured 14 × 12 × 10 cm and the cervix measured 4 × 3 cm. The myometrium contained multiple nodules consistent with intramural, submucosal and subserosal leiomyomas. The endometrium was smooth without anomaly. Microscopic examination of the myometrium revealed long fascicles of spindle cells without nuclear atypia and eosinophilic cytoplasm, few mitotic figures < 1/10 (at × 400) and no tumour necrosis. The endometrium and the cervix were normal. The aspect was in favour of multiple benign smooth muscle tumours (uterine leiomyomatosis). No leiomyosarcoma or neurofibroma was diagnosed.

## Discussion

Leiomyomas, which are benign smooth muscle cell tumours, are the most common uterine neoplasms. Approximately 20-30% of women over 35 years with uterine leiomyomas have clinical manifestations (menorrhagia, pelvic pain, pressure) [1]. Extrauterine leiomyomas are much less frequent and may be located at any anatomic site including the genitourinary tract (vulva, ovaries, urethra, and urinary bladder) and the gastrointestinal tract [1].

We report here a case of uterine leiomyomatosis in a 47-year-old patient with NF1 that raised some diagnostic issues. The lesions were asymptomatic until the patient presented within a year of her last examination with a highly suspect, tender abdominal and pelvic mass. Moreover, one of the lesions displayed focal FDG uptake, suggesting a potential neoplastic lesion. However, MRI confirmed that the lesion was a leiomyoma with abundant cellularity. This situation is uncommon but not rare in pre- and post-menopausal women. Abundant cellularity and hormonal dependency without malignant transformation are responsible for such uptake [4,5]. Another question raised by the PET/CT scan results was whether or not these lesions were NFBs or even a malignant transformation of a uterine NFB. Unfortunately, the PET/CT scans demonstrated varying degrees of FDG uptake in benign NFBs [6]. Therefore, PET/CT may not be a useful discriminating exam in this peculiar gynaecologic situation. Of note, our patient did not have any indication of a familial history of cutaneous and uterine leiomyomatosis [7].

At first glance, the association of uterine leiomyoma and NF1 may seem coincidental, as the first condition is frequent in the general population. However, there are several reports suggesting a true link between leiomyoma and NF1. The *NF1 *gene is a tumour suppressor gene located on chromosome 17 that encodes neurofibromin, a widely expressed negative regulator of intracellular RAS signalling in various tissues. Patients with NF1 are prone to develop various tumours. Case reports of gastrointestinal leiomyomas in patients with NF1 have been described on a regular basis in the literature since the fifties [2,8,9]. Patients are usually diagnosed with multiple tumours of the small intestine in a context of anaemia and gastrointestinal bleeding [2,10]. In addition, anecdotal cases of leiomyma and leiomyosarcoma of the urinary tract have been reported [11,12]. To the best of our knowledge, only Mellor Pita *et al *reported a case of a 40-year-old woman with NF1 and an abdominal mass that revealed both uterine and gastric leiomyomas [13]. Moreover, Wei *et al *reported the case of a 39-year-old woman with a plexiform NFB of the uterus associated with adenomyosis and uterine leiomyoma [14]. The female genital system is rarely affected during NF1 [14]. NFBs are most commonly located on the vulva, but other locations such as the clitoris, vagina, myometrium and ovary have occasionally been reported. There are also very few cases of uterine cervical NFBS [14]. Differential diagnoses of uterine NFB include schwannoma, neuroma, leiomyoma and myxoma [14].

## Conclusion

We here report a new case of uterine leiomyomatosis during NF1. This condition may be under-recognised because uterine leiomyomas are frequent in the general population. Physicians should keep in mind that female patients with NF1 presenting nonspecific symptoms such as menorrhagias, pelvic pain, discomfort and pressure should be examined for pelvic conditions including neurofibromas and leiomyomas [14,15]. In our opinion, the true incidence of uterine leiomyoma associated with NF1 deserves to be explored in a larger series of female patients with NF1, given the previously reported cases of NF1-associated leiomyoma of the gastrointestinal and urinary tracts.

## Consent

Written informed consent was obtained from the patient for publication of this case report.

A copy of the written consent is available for review by the Editor-in-Chief of this journal.

## Competing interests

The authors declare that they have no competing interests.

## Authors' contributions

NK and BG made major contributions to conception and design, acquisition of data, analysis and interpretation of data and drafting the manuscript. HP performed the pathological examination of the leiomyomas and was involved in drafting the manuscript and critically revising it for important intellectual content. All authors read and approved the final manuscript.

## References

[B1] Fasih N Prasad Shanbhogue AK Macdonald DB Fraser-Hill MA Papadatos D Kielar AZ Doherty GP Walsh C McInnes M Atri M Leiomyomas beyond the uterus: unusual locations, rare manifestations Radiographics 2008 28 1931 48 10.1148/rg.287085095 19001649

[B2] Cox JG Royston CM Sutton DR Multiple smooth muscle tumors in neurofibromatosis presenting with chronic gastrointestinal bleeding Postgrad Med J 1988 64 149 151 3140227 10.1136/pgmj.64.748.149 PMC2428782

[B3] Schmid RA Schöb OM Klotz HP Vogt P Weder W VATS resection of an oesophageal leiomyoma in a patient with neurofibromatosis Recklinghausen Eur J Cardiothorac Surg 1997 12 659 62 10.1016/S1010-7940(97)00210-8 9370414

[B4] Nishizawa S Inubushi M Kido A Miyagawa M Inoue T Shinohara K Kajihara M Incidence and characteristics of uterine leiomyomas with FDG uptake Ann Nucl Med 2008 22 803 10 10.1007/s12149-008-0184-6 19039559

[B5] Khademi S Westphalen AC Webb EM Joe BN Badiee S Hawkins RA Coakley FV Frequency and etiology of solitary hot spots in the pelvis at whole-body positron emission tomography/computed tomography imaging Clin Imaging 2009 33 44 8 19135929 10.1016/j.clinimag.2008.06.026 PMC2743966

[B6] Son JM Ahn MI Cho KD Yoo J Park YH Varying degrees of FDG uptake in multiple benign neurofibromas on PET/CT Br J Radiol 2007 80 e222 6 10.1259/bjr/31510627 17928494

[B7] Marque M Avril MF Bressac de Paillerets B Guillot B Richard S Bessis D Familial cutaneous and uterine leiomyomatosis Ann Dermatol Venereol 2008 135 612 6 10.1016/j.annder.2008.04.010 18789302

[B8] Ishizaki Y Tada Y Ishida T Bandai Y Idezuki Y Hitoshi N Mitio I Leiomyosarcoma of the small intestine associated with von Recklinghausen's disease: report of a case Surgery 1992 111 706 710 1595066

[B9] River L Silverstein J Tope JW Benign neoplasm of the small intestine: a clinical comprehensive review with reports of 20 new cases Int Surg 1956 102 1 38 13299024

[B10] Chu MH Lee HC Shen EY Wang NL Yeung CY Chen BF Shih SL Gastro-intestinal bleeding caused by leiomyoma of the small intestine in a child with neurofibromatosis Eur J Pediatr 1999 158 460 2 10.1007/s004310051120 10378392

[B11] Däuth TL Conradie M Chetty R Leiomyoma of the bladder in a patient with von Recklinghausen's neurofibromatosis J Clin Pathol 2003 56 711 2 12944559 10.1136/jcp.56.9.711 PMC1770053

[B12] Poleksic S Leiomyosarcoma of urinary bladder in von Recklinghausen's neurofibromatosis Urology 1977 10 341 2 10.1016/0090-4295(77)90164-9 411206

[B13] Mellor Pita S Yebra Bango M González Hernando C Ramón y Cajal S Von Recklinghausen's disease and abdominal mass Rev Clin Esp 1999 199 759 60 10638244

[B14] Wei EX Albores-Saavedra J Fowler MR Plexiform neurofibroma of the uterine cervix: a case report and review of the literature Arch Pathol Lab Med 2005 129 783 6 15913429 10.5858/2005-129-783-PNOTUC

[B15] McLucas B Diagnosis, imaging and anatomical classification of uterine fibroids Best Pract Res Clin Obstet Gynaecol 2008 22 627 42 10.1016/j.bpobgyn.2008.01.006 18328787

